# Human Papilloma Virus and Autophagy

**DOI:** 10.3390/ijms19061775

**Published:** 2018-06-15

**Authors:** Domenico Mattoscio, Alessandro Medda, Susanna Chiocca

**Affiliations:** 1 Department of Medical, Oral, and Biotechnology Science, University of Chieti-Pescara, 66100 Chieti, Italy; 2 Center on Aging Science and Translational Medicine (CeSI-MeT), University of Chieti-Pescara, 66100 Chieti, Italy; 3 Department of Experimental Oncology, European Institute of Oncology, 20139 Milan, Italy; alessandro.medda@ieo.it

**Keywords:** HPV, autophagy, cervical cancer, head and neck cancer, viral tumorigenesis, oncoviral proteins

## Abstract

Human papilloma viruses (HPVs) are a group of double-stranded DNA viruses known to be the primary cause of cervical cancer. In addition, evidence has now established their role in non-melanoma skin cancers, head and neck cancer (HNC), and the development of other anogenital malignancies. The prevalence of HPV-related HNC, in particular oropharyngeal cancers, is rapidly increasing, foreseeing that HPV-positive oropharyngeal cancers will outnumber uterine cervical cancers in the next 15–20 years. Therefore, despite the successful advent of vaccines originally licensed for cervical cancer prevention, HPV burden is still very high, and a better understanding of HPV biology is urgently needed. Autophagy is the physiological cellular route that accounts for removal, degradation, and recycling of damaged organelles, proteins, and lipids in lysosomal vacuoles. In addition to this scavenger function, autophagy plays a fundamental role during viral infections and cancers and is, therefore, frequently exploited by viruses to their own benefit. Recently, a link between HPV and autophagy has clearly emerged, leading to the conceivable development of novel anti-viral strategies aimed at restraining HPV infectivity. Here, recent findings on how oncogenic HPV16 usurp autophagy are described, highlighting similarities and differences with mechanisms adopted by other oncoviruses.

## 1. Introduction

Oncovirus infection represents one of the leading causes of cancer worldwide, accounting for approximately 12% of total cancer burden [1]. Among them, human papilloma virus (HPV) infection accounts for approximately 2% and 7% of the total cancer burden in more developed and less developed countries, respectively [2]. In particular, high risk (HR) HPVs, such as HPV16, are the main cause of cervical cancer and are also involved in other anogenital tumors, in non-melanoma skin cancers and head and neck cancer (HNC) [3]. Even if vaccines against common tumorigenic HPVs have been established, the proportion of HPV-related malignancies, in particular HNC, is steadily increasing [4]. For these reasons, and for the absence of targeted therapies to treat HPV-tumors, a deep understanding of the molecular mechanisms underlying HPV tumorigenesis is fundamental.

Autophagy is a self-consumption mechanism occurring inside the cell mediating degradation of cargoes from different origins by lysosomal activity. In particular, autophagy is involved in degradation of long-lived proteins, but also of organelles like mitochondria (mitophagy), or endoplasmic reticulum (ER-phagy). Aggrephagy encompasses the degradation of aggregate-prone intracytoplasmic proteins, such as α-synuclein, mutant huntingtin, and protein tau. Xenophagy entails the degradation of pathogens, such as bacteria and viruses, that escape or cause ruptures in the vacuoles where they are confined (for recent reviews on these topics see [5,6,7,8]).

Depending on the delivery route carrying materials for digestion, autophagy can be distinguished in chaperone-mediated autophagy, microautophagy, and macroautophagy. In chaperone-mediated autophagy molecular chaperones translocate unfolded proteins containing a specific sequence in the cytosol into lysosomes by interaction with lysosome-associated membrane protein (LAMP) type 2A, which acts as a receptor (reviewed in [9]). Microautophagy is characterized by a direct invagination of lysosomal membrane with engulfment of cytoplasmic material (reviewed in [10]). In macroautophagy (hereafter simply referred as autophagy) a double-membraned organelle, the autophagosome, sequesters cytosolic materials, then fuses with a lysosome, resulting in an autolysosome, where cargoes are digested by lysosomal hydrolases (reviewed in [11]). Autophagy is a complex mechanism that can be schematically divided into phagophore initiation, elongation, and fusion with lysosomes. In mammals, during the initiation phase, the unc-51 like autophagy activating kinase 1 (ULK1) kinase complex, which consists of ULK1 (or ULK2), autophagy-related protein 13 (ATG13), FAK family kinase-interacting protein of 200 kDa (FIP200), and ATG101, is recruited either on cellular membranes at the ER-mitochondrial junction [12] or at the ER-Golgi intermediate compartment [13]. The activity of the complex is tightly regulated by nutrient conditions through the mammalian target of rapamycin (mTOR) [14]. In particular, during starvation, low energy levels are sensed by the AMP activated protein kinase (AMPK), which phosphorylates mTOR and inactivates the mTOR1 complex (mTORC1). Inactive mTORC1 dissociates from the ULK1 kinase complex, resulting in activation of ULK1 which phosphorylates mammalian ATG13 (mATG13) and FIP200 leading to phagophore initiation. The sequential recruitment of autophagy factors and membranes produces the phagophore, a curved, double-membrane sheet that detaches from the membrane it originates. Then, during elongation, the class III phosphatidylinositol 3 kinase (PI3K) complex formed by the vacuolar protein sorting 34 (VPS34) PI3K, its regulatory subunits ATG14L, VPS15, and Beclin 1, mediate phagophore membrane expansion. In particular, Beclin 1 dissociation from B-cell lymphoma/leukemia-2 (Bcl-2) activates the class III PI3K complex, leading to the recruitment of two ubiquitin-like conjugation systems that promote the conjugation of lipid molecules, namely phosphatidylethanolamine (PE), to the microtubule-associated protein 1 light chain 3 (LC3) (for a detailed review of autophagosome biogenesis see [15]). Lipidated LC3 (LC3-II), localized on both sides of the nascent autophagosome, is a specific marker of autophagosome formation widely used as an indicator of autophagic flux, and it regulates membrane elongation and autophagosome maturation [16]. Finally, the activity of the class III PI3K complex II, consisting of VPS34, VPS15, Beclin 1, and UV radiation resistance-associated gene protein (UVRAG), stimulates the Ras-associated protein-7 (Rab7), inducing its GTPase activity and leading to autophagosome fusion with late lysosomes [17]. This results in autolysosomes formation, with degradative properties given by lysozymes.

Although initially believed to have a role only in response to starvation and in degradation and recycling of macromolecules and organelles, it is now clear that autophagy impacts a wide range of cellular functions both in health and disease. For example, autophagy has important pathogenetic roles in muscular disorders, neurodegeneration, aging, and in inflammatory conditions (reviewed in [11]). Altered autophagy is also a key driver of malignant transformation. Indeed, autophagy could have a dual role in tumor development: tumor suppressor in early stages by restricting inflammation and intracellular reactive oxygen species (ROS) levels and supporting genomic stability and integrity, whilst tumor promoter in later stages of tumorigenesis through the increase in cancer cells’ ability to live in nutrient starvation and hypoxic environment (recently reviewed in [18]). Therefore, in many cancer types, autophagy is inhibited in pre-cancerous cells while activated in growing tumors. In addition, given the pleiotropic role for autophagy in regulating a variety of cellular activities, it is not surprising that some pathogens evolved different mechanisms to usurp autophagic machinery to completely subvert the host milieu to their own advantage. Viruses, in particular, can evade autophagy in different ways, including prevention of autophagy induction, autophagosome maturation, pathogen recognition, and using autophagy components to increase their ability to survive or to replicate (reviewed in [19]).

Here, we focus on the role of autophagy during HPV infection and tumorigenesis and its potential function as a target for development of novel anti-viral and anti-neoplastic agents, also discussing similarities and differences on mechanisms adopted by other oncoviruses in order to modulate the host autophagic response.

## 2. HPV Manipulation of Host Autophagy

In recent years, a close interplay between autophagy and HPV has emerged. Like many other viruses, HPV also manipulates the autophagic machinery to promote its lifecycle inside host-infected cells. Additionally, oncogenic HPVs exploit cellular autophagy to support proliferation of infected epithelial cells, significantly contributing to cancer progression. In the following sections we will discuss how autophagy affects HPV pathogenesis and the strategy adopted by the virus to exploit this process in order to improve its persistence in the host.

### 2.1. Autophagy Inhibition Promotes HPV Infectivity

HPV is a double-stranded DNA virus with a circular genome that encodes early-, including *E1*, *E2*, *E4*, *E5*, *E6*, and *E7* essential for replication, transcription and transformation, and late-genes *L1* and *L2*, encoding for viral capsid proteins. The replication cycle of HPV is tightly linked to differentiation of the infected epithelium. Indeed, while HPV binding and infection arise exclusively in basal keratinocytes at microtrauma sites likely due to sexual intercourse, viral protein production and virus assembly occurs only in the upper differentiated layers of the epithelia. In wound basal layer, HPV particles initially interact with the basement membrane mostly through heparan sulfate proteoglycans (HSPGs)—capsid L1 contacts, and subsequently bind to HSPGs present on basal keratinocytes cell surface. This attachment triggers conformational changes in the L2 capsid protein, resulting in exposure of a consensus cleavage site in L2 N-terminus, whose proteolysis facilitates further interaction of viral capsid with secondary receptor(s) present on keratinocytes membrane. After such binding, HPVs are generally internalized by clathrin-dependent endocytosis, which is initially reliant on actin-rich cell protrusions, acting as the transport mechanism along the endocytic machinery (reviewed in [20]).

Both binding and internalization of HPV particles are processes intimately linked to manipulation of host autophagy. Interaction with HPSGs triggers rapid activation of several signaling pathways in host cells that benefit HPV infection and, among them, HPV entry is associated with autophagy suppression through activation of the mTOR pathway. In particular, upon HPSGs binding, HPV16 interacts with epidermal growth factor receptors (EGFRs) present on the plasma membrane of target cells [21], resulting in protein Kinase B (Akt) phosphorylation and inactivation of phosphatase and tensin homolog (PTEN), culminating with phosphorylation and activation of mTOR [22]. Once activated, mTOR promotes phosphorylation and activation of the mTOR complex 1 (mTORC1), substrates 4E-BP1 (elongation initiation factor (EIF)-4E binding protein 1) and S6K1 (ribosomal protein S6 kinase 1) [14], key components of the translation machinery, while it phosphorylates and de-activates ULK-1, the kinase localized on isolation membranes that is involved in autophagosome nucleation [23]. Thus, activation of the mTORC1 complex through manipulation of the PI3K/Akt/mTOR pathway, promoted by HPV binding, increases protein synthesis and inhibits cellular autophagy at very early stages of HPV16 cycle, namely before virus entry in target cells. Both of these functions are needed for successful infection of human keratinocytes [22]. Consistently, autophagy impairment obtained with the early inhibitor 3-methyladenine (3-MA) [24], or through genetic ablation of essential autophagic genes, greatly increases HPV16 infectivity in human keratinocytes [22,25,26], strongly indicating the importance of host autophagy in patrolling the early steps of HPV lifecycle. Mechanistically, rather than modulating virus attachment or internalization, autophagy inhibition protects HPV16 capsid degradation inside the autophagosome [26] through delaying the rate of L1 digestion [25].

**Figure 1:**
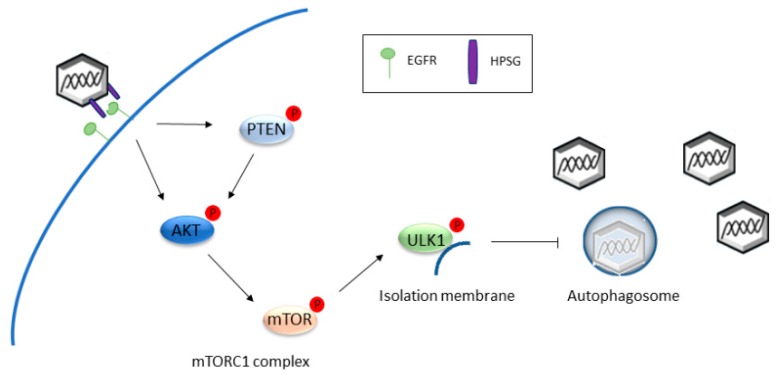
HPV16 binding and internalization inhibits autophagosome formation. HPV virions decorated with HPSGs interact with EGFRs present on the plasma membrane of target cells, resulting in Akt and PTEN phosphorylation, and in phosphorylation and activation of mTOR. Activated mTOR phosphorylates and inactivates ULK1, present on isolation membranes, inhibiting autophagosome nucleation and, therefore, delays L1 digestion and capsid degradation inside autophagosomes. Arrows indicate activating pathways; T-bar indicates inhibitory pathway; blue lines represent cellular and isolation membranes.

Therefore, HPV16 diminishes the host-autophagic response, exploiting molecular events promoted by its binding and internalization as a defense mechanism to protect incoming virions from rapid degradation, thus extending HPV’s lifespan inside infected cells (Figure 1).

### 2.2. HPV16 Dampens Autophagy to Promote Cancer Progression

Once internalized in basal cells, HPV traffics via the endosomal compartment where capsid proteins are degraded inside acidified endosomes, and the viral genome enters the nucleus (reviewed in [27]). Here, HPV DNA is amplified and maintained as episomes in basal cells of the epithelium by E1- and E2-mediated mechanisms. Infection may latently persist or the virus may be triggered to proliferate, relying on keratinocyte replication proteins that mediate viral DNA synthesis. The infected basal cell initially enters into the suprabasal layer where viral episomal gene expression is maintained at low levels, and finally in the granular and cornified layers where the keratinocyte differentiates, exits the cell cycle, drives viral DNA replication, and promotes the expression of *E4*, *E5*, *E6*, *E7*, *L1*, and *L2* genes, leading to HPV virion formation and release (recently reviewed in [28]).

Although HR HPV infection is an important etiological factor for several cancer types, its presence is necessary, but not sufficient, for cancerogenesis, indicating that other events (such as genomic instabilities or failure of the immune system) are needed for cancer to occur. To this end, a fundamental role in cell transformation is mediated by E5, E6, and E7 oncoproteins, able to modify cell-cycle regulation, induce human telomerase reverse transcriptase (hTERT) expression with consequent telomere maintenance, and block apoptosis [29]. As a result, DNA damage and mutations accumulate, leading to the formation of a transformed cell, koilocyte [30], and then to the development of cancers. In particular, E5 is a multifunctional protein that contributes to cellular transformation mainly associating with and enhancing signaling network of growth factor receptors (reviewed in [31]). E6 promotes the proteasomal degradation of the tumor suppressor p53, through the ubiquitin ligase E6AP [32]. E7 interacts with the pocket proteins (retinoblastoma-RB, p107, and p130) family involved in cell cycle progression [33]. In addition, viral oncogenes can alter a large number of other cellular proteins (reviewed in [29]), modifying their normal function and driving transformation of epithelial basal and parabasal cells in koilocytes. However, before malignant transformation, the persistent activity of HPV oncoproteins can establish a number of precancerous lesions classified as low-grade squamous intraepithelial lesions (LSILs), and high-grade SILs (HSILs) during cervical cancer progression. LSILs are characterized by abnormalities in the lower third of epithelium, and lesions can spontaneously regress or evolve into HSILs. Transformed cells are present in HSILs throughout the epithelium and HPV more likely persists in the host also integrating its DNA in the cellular genome, contributing to cancer progression [34].

**Figure 2:**
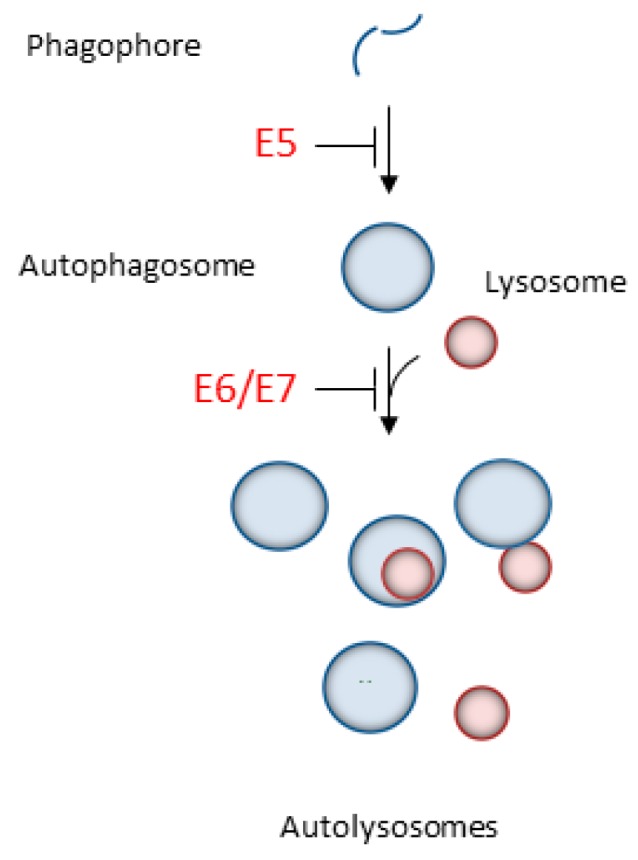
HPV16 oncoproteins dampen the host autophagic response acting at different levels of the autophagic pathway. E5 interferes with the transcriptional activation of the autophagic machinery down-regulating *Beclin 1*, *ATG5*, *LC3*, *ULK1*, *ULK2*, *ATG4a*, and *ATG7* mRNAs, thus suggesting inhibition of phagophore assembly, while E6 and E7 inhibit autophagosome/lysosome fusion may be due to depletion of autophagic genes. Arrows indicate activating pathways; T-bars indicate inhibitory pathways.

In addition to the well-characterized pathways that ultimately mediate HPV-promoted cellular transformation, a significant role for autophagy during the carcinogenic process is now emerging. Notably, the fact that E5, E6, and E7 oncoproteins evolved diverse mechanisms to affect the host autophagic pathway to induce cellular transformation underlines the importance of autophagy during each step of viral-mediated tumorigenesis (Figure 2). In particular, ectopic expression of HPV16 E5 in an HPV-negative keratinocytic cell line reduced levels of the autophagosome marker LC3-II, prevented the degradation of the autophagic susbstrate p62, and diminished the number of autophagosomes in the keratinocyte growth factor (KGF) and serum-starved triggered cells, indicating failure in autophagosome assembly. Consistently, depletion of E5 from HPV-positive cells abrogated these effects confirming that E5 expression drives autophagy inhibition acting at very early steps of the autophagic pathway. Mechanistically, HPV16 E5 interferes with the transcriptional activation of the autophagic machinery, down-regulating mRNA levels of key autophagic genes, such as *Beclin 1*, *ATG5*, *LC3*, *ULK1*, *ULK2*, *ATG4a*, and *ATG7* [35], suggesting inhibition of phagophore assembly. Notably, opposite to E5, which prevents autophagosome formation, HPV16 E6/E7 dampens autophagy, affecting one of the later steps (autophagosome-lysosome fusion), thus providing another mechanism against autophagy progression during HPV tumorigenesis. HPV16 E6/E7 overexpression in primary human keratinocytes increased the expression of both the lipidated LC3 and p62, indicating autophagosome accumulation (increase in LC3-II) in spite of decreased degradation capability (increased p62). Additionally, confocal and electron microscopic analyseis revealed reduced autolysosome formation in E6/E7 cells, pinpointing the fusion between autophagosomes and lysosomes as the defective stage affected by these viral oncoproteins. Impairment in later steps of autophagy was mainly due to an E6/p53-dependent mechanisms since HPV16 E6 expression alone increased both LC3-II and p62, and, compared to E6 wild type, E6 mutants defective in p53 degradation have a diminished ability to increase p62 expression in combination with E7 [36]. In addition, HPV16 E7 expression is also able to increase LC3-II levels in keratinocytes [37], suggestive of autophagic defects. Consistently with these results, depletion of the bicistronic HPV16 *E6*/*E7* mRNA using siRNA targeted against the E7 sequence induced significant enrichment of autophagic genes and phenotypic evidence of autophagy activation, such as the appearance of autophagosomes, punctate expression of LC3, conversion of LC3-I to LC3-II, and reduced levels of the autophagy substrate p62 [38].

In support of the above described in vitro results collectively indicating that HPV16 oncoviral proteins impair autophagy through different mechanisms, a number of other observations obtained in vivo or ex vitro confirmed that autophagy manipulation is an important co-factor of several HPV-derived malignancies. These results also highlight several autophagic biomarkers that could be used for diagnosis and to monitor the evolution of diseases (Table 1). In fact, anuses of dysplastic HPV16 transgenic mice (K14-E6/E7) are characterized by an amplified punctate expression of LC3 and p62, and an increase in autophagosomes without evidence of autolysosomes, as shown by transmission microscopy and immunofluorescence analysis. This peculiar pattern of autophagic markers expression, indicating autophagy inhibition at late stages, was also found in human samples, confirming that autophagic dysregulation is a key determinant of HPV-associated anal carcinogenesis [39]. In support of this hypothesis, treatment of both WT and K14-E6/E7 mice with the late autophagic inhibitor chloroquine significantly increases anal cancer development [39,40], while autophagy activation reduces tumor onset [40].

Similarly to anal cancer, autophagy impairment is also relevant for cervical cancer, the most common HPV-induced malignancy. Compared to adjacent normal tissues, p62 expression gradually increased through dysplastic transformation of cervical tissues from LSIL to HSIL, indicating that autophagy impairment during lesion progression could be clinically relevant to the HPV-mediated carcinogenic process [36]. Consistently, opposite to p62, Beclin 1 expression decreases during cancer progression since its levels were found to be significantly lower in dysplastic cervix and even less in cervical cancer specimens, still confirming a progressive dampening of the autophagic response during HPV-mediated cervical transformation. Notably, Beclin 1 levels, as determined by immunohistochemistry, negatively correlated with cervical cancer differentiation, lymph node metastasis, recurrence, death [41], and histological grade [42], indicating the fundamental role of autophagy in cancer development. In addition to Beclin 1, LC3 amount also negatively correlates with HR HPVs infection and higher clinical tumor node and lymph node metastasis [44], again indicative of a reduced autophagic flux in HPV-infected cervical tissues and worse clinicopathological features in affected patients. Moreover, the expression of the anti-autophagy protein ATAD3A in cervical cancer specimens is positively associated with persistent HPV infection, FIGO stage, lymph node involvement, proinflammatory and metastasis-related cytokines, and patient survival. In addition, ATAD3A silencing increases autophagosomes number and markedly decreases drug resistance in uterine cervical cancer cells, further underlying the importance of autophagy in HPV-mediated cancer development [45].

In addition, to hijack host genes and proteins for their own benefit, HR HPVs impact on micro RNAs (miRNAs) expression to dampen cellular autophagy and facilitate tumor progression. In fact, miRNAs profile of cervical tissues infected by HR HPVs showed increased expression of miR-224-3p, in dysplastic and cancer tissues. Gain and loss of function approaches in cervical cancer cell lines evidenced that miR-224-3p regulates autophagy through alteration of FIP200 levels, a protein involved in autophagosome formation [47]. Therefore, HPVs manipulate host autophagy by reducing the expression of FIP200 through the overexpressed miR-224-3p [43]. Similarly, miR-155-5p expression was recently found to be significantly down-regulated in cervical cancer tissues. Suppression of miR-155-5p levels in HPV positive cell lines inhibit LC3 while promotes p62 protein expression, indicative of autophagic defects, through a pyruvate dehydrogenase kinase 1 (PDK1)/mTOR-dependent mechanism [46].

Collectively, the above described findings highlighted that HR HPVs extensively impact on autophagy through many different mechanisms with the common endpoint of quenching cellular autophagic response. Therefore, manipulation of host autophagy is a crucial regulator of HPV-mediated malignant transformation and could be rationally targeted to develop innovative drugs, and exploited to generate useful biomarkers to monitor the clinical outcome of HPV-related diseases.

### 2.3. Tumor Viruses Usurp Host Autophagy during Their Infection Cycle

Similarly to HPV16, other oncoviruses affect the cellular autophagic response, suggesting the importance of the autophagic route in mediating oncovirus persistence and tumorigenesis. For example, the Epstein-Barr herpes virus (EBV), associated with several tumors, such as Burkitt’s lymphoma, nasopharyngeal carcinoma, and Hodgkin’s disease, decreases autophagy during its lytic replication cycle, and this activity promotes viral infectivity. Autophagy inhibition also increased EBV lytic gene expression, intracellular viral DNA and viral progeny yield [48]. Likewise to HPV16 E6/E7 [36], EBV promotes autophagosomes accumulation due to a reduction in autophagosome-lysosome fusion in a Rab7-dependent manner [49]. The block in the final degradative step allows the virus to prevent autophagy-mediated viral degradation, thus extending EBV infection and cancer progression. Moreover, the human T-cell leukemia virus type 1 (HTLV-1), a member of the deltaretrovirus family known to cause adult T-cell leukemia mainly due to the action of the oncoprotein Tax [50], also manipulates autophagy during its tumorigenic cycle to increase its persistence, promote proliferation, and inhibit apoptosis of infected T lymphocytes [51,52]. HTLV-1 infection induced autophagosome accumulation through the action of the viral protein Tax. Tax protein acts on autophagosome accumulation by blocking autophagosome-lysosome fusion [51], through the exploitation of peculiar cellular mechanisms involving recruitment of autophagic molecules to lipid rafts in a NF-κB inhibitor kinase (IKK) complex-dependent manner [52,53].

In addition, another oncogenic virus belonging to the herpes virus family, the Kaposi sarcoma herpesvirus (KSHV) associated with Kaposi sarcoma, a form of multicentric Castleman disease, and primary effusion lymphoma [54], also suppresses host autophagy through a mechanism involving the viral protein FADD-like interferon converting enzyme or caspase 8 (FLICE) inhibitory protein (vFLIP) -mediated dysregulation of the IKK/NF-κB (nuclear factor kappa-light-chain-enhancer of activated B cells) signaling axis [55,56]. This effect, occuring during virus latency, blocks senescence, and facilitates the proliferation of KSHV-infected cells [56]. During lytic reactivation of latent virus, the replication and transcription activator protein (RTA) potently increases autophagy of infected cells, and autophagy inhibition at this stage affects RTA-mediated lytic gene expression and viral DNA replication [57]. Therefore, the timely and coordinated usurpation of host cellular autophagy is fundamental to trigger completion of KSHV infection cycle.

Opposite to HPV, Hepatitis B virus (HBV), a hepatotropic virus that can cause hepatocellular carcinoma (HCC) [58], enhances the autophagic response of infected liver cells, transgenic mouse models, and tissue patients [59,60] to positively modulate HBV DNA replication. Mechanistically, the multifunctional Hepatitis B virus X protein (HBx) interacts with VPS34 leading to the activation of the PI3K complex III and promotion of autophagosome formation [59], and via Beclin-1 phosphorylation through a death-associated protein kinase (DAPK)-dependent manner [61]. However, despite autophagy induction, HBV infection does not alter the rate of autophagic degradation, indicating a later impairment of the lysosome-digestive properties. In fact, HBx could alter Rab7 expression and the V1D subunit of the proton-pumping V-type ATPase (V-ATPase) activity, resulting in a reduced maturation and function of lysosomes [62,63]. In addition to the induced production of HBV virions through autophagy activation, HBx also protects HBV-infected cells from apoptosis exploiting autophagy. To this end, HBx acts as an autophagic adaptor that facilitates the recruitment of dead receptor tumor necrosis factor receptor superfamily member 10B (TNFRSF10B) to the autophagic machinery, promoting its degradation and, thus, dampening the recognition of infected cells by immune cells. In addition, silencing of essential autophagic proteins, such as ATG5, ATG12, and ATG16L1, impairs HBV formation and release, altering the intracellular distribution of HBV. HBV core interaction with ATG12, a component of the autophagic elongation complex, drives HBV association with cellular membranes and allows the formation of mature viral particles [64], further underlying the importance of autophagy in HBV infection.

Similarly to HBV, the related Hepatitis C virus (HCV) is also able to affect autophagy to increase virus growth and survival in infected cells. Specifically, HCV induces autophagosome formation through a double mechanism: inducing a time-dependent dephosphorylation of ULK1 [65] and up-regulating transcriptional levels of Beclin 1 [66]. However, promotion of autophagosome formation occurs together with the inhibition of autophagosome maturation, mediated by over-expression of Rubicon (RUN domain and cysteine-rich domain containing, Beclin 1-interacting protein), a negative regulator of UVRAG which inactivates the UVRAG-PI3KC3 complex and Rab7 [67], and dislocation of V-ATPase [68]. Since HCV associates with lipid rafts inside autophagosome membranes to improve replication of its RNA [69] while autolysosomes would promote degradation of this replication complex, HCV manipulates host autophagy to improve its own replication. Moreover, autophagy manipulation also allows HCV release in the extracellular space, thus enabling mature HCV egression from infected cells and viral transmission [70].

## 3. Conclusions

Autophagy is a physiological pathway that mediates degradation of macromolecules and damaged structures, playing a key role in maintaining homeostasis, regulating crucial cellular functions, and mediating stress responses. Therefore, it is not surprising that a number of viruses evolved ingenious strategies to manipulate the host autophagic response in order to subvert cellular activities and to create a more conducive environment for viral replication [71]. Among them, a particularly important role for autophagy in mediating oncovirus infection, persistence, and tumorigenesis arose (reviewed in [72]). Recently, HPV16 has also emerged as an oncogenic virus with the ability of diverting cellular autophagy during its lifecycle, and significant evidence indicates that viral proteins dampen the autophagic response through many different mechanisms acting at each step of viral infection and tumorigenesis.

HPV16 impacts on autophagy both during the initial steps of infection (namely, adhesion and entry of viral particles into target cells) to avoid early clearance of viral particles by autophagic digestion (Figure 1), and later to help in promoting transformation of infected cells throughout the carcinogenic process (Figure 2, Table 1). Of note, the three different oncoviral proteins E5, E6, and E7 adopt different strategies to turn off host autophagy, further underlying how manipulation of the autophagic pathway is fundamental to finally promoting malignant transformation. Mechanistically, despite many different cellular pathways being affected by viral oncoproteins from diverse oncogenic viruses, a common outcome that seems to emerge from comparative analysis is the inhibition of the final autophagic step, namely autophagosome-lysosome fusion, as the main target of autophagy inhibition. EBV, HTLV-1, HBV, and HCV impair autolysosome formation, suggesting a deleterious role for autophagic degradation in oncovirus infection. Consistently, HPV16 also specifically dampens this step. Hence, given the importance of autolysosome formation on oncovirus tumorigenesis, and the potential of targeting this step for therapeutic purposes, more studies are necessary to better clarify the connection between HPV and autophagy, and to identify novel autophagy-based molecular targets for biomarker validation (Table 1) and/or drug development. To this end, the recently-characterized autophagic inducers able to increase autophagosome-lysosome fusion [73] could represent an important starting point to identify putatively useful molecules and to suggest relevant clinical treatment strategies against HPV infections.

In addition, even if most of the reported in vitro evidence has been obtained using HPV16 virions or oncoviral proteins, it could be very important to determine similarities and divergences in the host autophagy impact among different HR HPV genotypes, as well as to determine the role of autophagy during low-risk HPV infection. These results will highlight and strengthen the use of approaches aimed at modulating autophagy during the course of the HPV cycle.

It is evident that autophagy inhibition promotes the HPV lifecycle and tumor progression and that strategies aimed at restoring the autophagic response during HPV infection and carcinogenesis could be of importance to restrain HPV-mediated diseases.

## Figures and Tables

**Table 1:** Autophagy alterations during HPV-mediated tumor progression. Up and down arrows indicate increased and decreased expression of the reported proteins, respectively. ATAD3A: ATPase family AAA domain containing 3A.

Tumor	Autophagic Protein Altered	Putative Use as Biomarker	Refs.
Anal dysplasia	LC3↑, p62↑	Diagnosis, progression	[39]
Cervical dysplasia	p62↑	Diagnosis, progression	[36]
	Beclin 1↓	Diagnosis, progression, prognosis	[41,42]
	miR-224-3p↑, FIP200↓	Diagnosis, progression	[43]
Cervical cancer	LC3↓	HPV infection, diagnosis, prognosis	[44]
	ATAD3A↑	HPV infection, diagnosis, prognosis, therapeutic predictivity	[45]
	miR-224-3p↑, FIP200↓	Diagnosis	[43]
	miR-155-5p↓	Diagnosis	[46]
